# Non-Obstetric Traumatic Vulvar Hematoma Managed in a Low-Resource Setting: A Case Report of a Rare Condition

**DOI:** 10.7759/cureus.40369

**Published:** 2023-06-13

**Authors:** Sowjanya Kurakula, Maheshwari Nallur Siddaraju, Varalakshmi Kumarswamy, Qurat ul Ain Raza, Abhijna Rao Kompella

**Affiliations:** 1 Obstetrics and Gynecology, Mamta Institute of Medical Sciences, Khammam, IND; 2 Obstetrics and Gynecology, Sekgoma Memorial Hospital, Serowe, BWA; 3 Obstetrics and Gynecology, Nyangabgwe Referral Hospital, Francistown, BWA; 4 Obstetrics and Gynecology, Gandhi Medical College, Musheerabad, IND; 5 Family Medicine, Medical Offices of Dr. Shaswathi Kale, San Jose, USA; 6 Obstetrics and Gynecology, RajaRajeshwari Medical College, Bangalore, IND; 7 Obstetrics and Gynecology, M.S Ramaiah Medical College, Bangalore, IND; 8 Obstetrics and Gynecology, RNT Medical Hospital, Udaipur, IND; 9 Internal Medicine, RAK Medical & Health Sciences University, Ras al Khaimah, ARE; 10 Obstetrics and Gynecology, Srinivasa Maternity and Nursing Home, Hyderabad, IND

**Keywords:** vulvar lesions, vulvar, vulvar pain, vulvar mass, trauma, perineal, massive hematoma, hematoma evacuation, hematoma expansion, hematoma

## Abstract

Most cases of vulvar hematomas are caused by either genital tract injury during childbirth or trauma. Although uncommon, instances of spontaneous vulvar hematomas occurring without trauma or unusual sexual practices have been reported. In this report, we present the case of a 24-year-old woman who experienced an injury after a fall, resulting in a rapidly enlarging vulvar hematoma. Due to the worsening pain and swelling, surgical intervention was undertaken for her management.

## Introduction

Vulvar hematomas are commonly observed and typically result from blunt trauma to the genitalia. They often arise from various causes, including puerperal injuries, physical assault, sexual intercourse, and straddle injuries [1]. In a study involving 20 women with non-obstetric traumatic vulvar hematoma, recent vulvar surgery was identified as one of the contributing factors [2]. The anatomical position of the vulvar region, combined with its ample vascularization through the internal pudendal artery, makes it prone to the formation of hematomas [3].

In non-obstetric trauma cases involving the female genitalia, approximately 30% of incidents are accompanied by urological injuries [4]. It is essential to conduct a thorough clinical examination of the urethra, and prioritizing urethral catheterization before initiating any repairs is crucial. Traumatic injuries to the vulva can vary in severity, ranging from minor to severe. Due to the abundant blood supply and the lack of valves in the perineal veins, the likelihood of significant hematomas is high. In severe cases, there is a potential risk of perforation into critical areas such as the peritoneal cavity, intestines, and bladder, which can pose life-threatening consequences [5].

## Case presentation

A 24-year-old multiparous woman presented to the emergency room with severe perineal pain and vaginal bleeding. The patient reported sustaining an injury from falling off a tree branch a few hours ago. Despite experiencing intense pain, she managed to walk, but with significant difficulty. The patient denied any substance abuse or history of sexual assault. She had attained menarche at age 13, and her last menstrual period had been 10 days ago. Her menstrual cycles were regular, lasting five days with a 30-day interval. She had had two vaginal deliveries in the past, and her last childbirth had been three years ago. She had no significant medical or surgical history and no known drug or food allergies.

**Figure 1:**
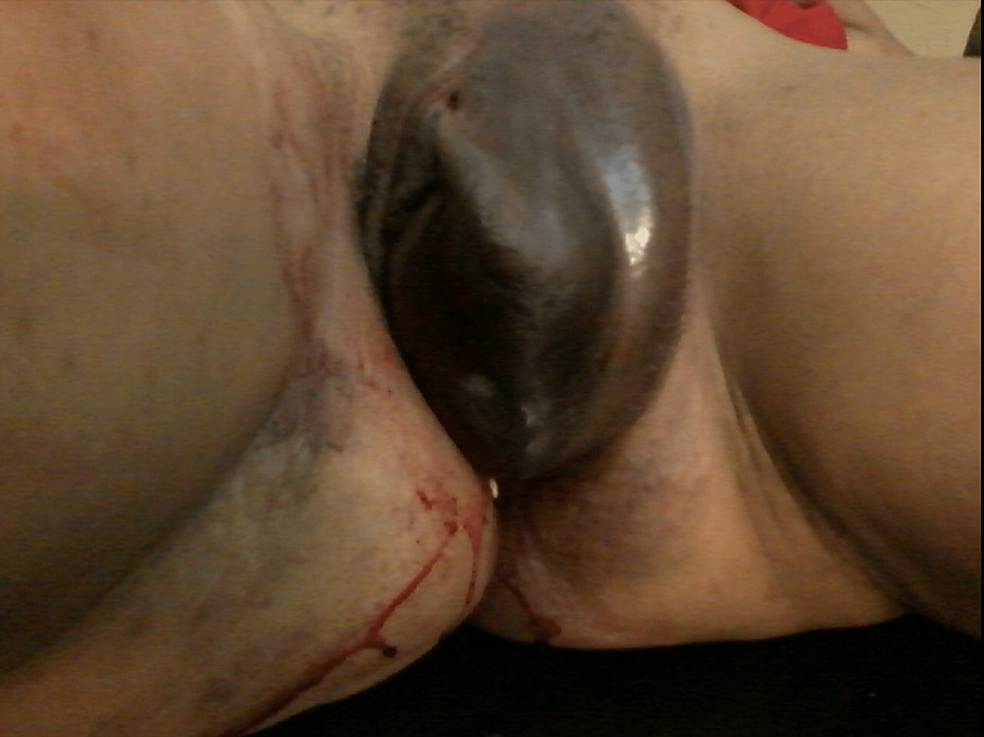
A large vulvar hematoma and bilateral contusions in the gluteal region

The patient managed to sit in a discomforting position, and her hip joint rotational movements were normal. She was afebrile but showed mild pallor. Her pulse rate was 98 beats per minute, and her blood pressure was 110/60 mmHg. Her heart sounds were within the normal range, and her lungs were clear on auscultation. Her abdomen was found to be soft without any organomegaly or free fluid. On genital examination, a rapidly expanding vulvar hematoma measuring 15 x 10 cm was observed, with scanty fresh blood oozing from the vagina (Figure 1). The overlying skin appeared tightly stretched. The hematoma was bluish in color, tense, tender, and warm to the touch. Contusions were present on both sides of the gluteal region, specifically around the gluteal tuberosities. Bilateral anterior pubic rami were intact and non-tender on palpation. Ringer lactate and normal saline infusion were initiated.

The preoperative assessments showed a hemoglobin level of 8 gm% and negative serological tests. The patient's hematological parameters, including platelet count, bleeding time, and clotting time, were in the normal range. Antiemetics and antibiotics were administered prophylactically before surgery.

Given the rapidly expanding hematoma, the patient was immediately shifted to the operating room, placed in a lithotomy position, and the perineum was cleaned and draped. Under saddle block spinal anesthesia, urethral catheterization was performed, and an incision was made over the most prominent area of the swelling, specifically at the mucocutaneous junction of the vulva. Approximately 750 ml of blood clots were drained, the bleeders were sutured, and cautery was used judiciously to control bleeding. The dead space was obliterated using 2-0 Vicryl sutures, and a 4 x 1-inch corrugated rubber drain was placed and secured. Hemostasis was achieved. The vaginal walls, cervix, and fornices were thoroughly inspected to exclude internal bleeding.

Postoperative assessments, including an X-ray of the pelvis and ultrasound of the abdomen, were normal. The patient received intravenous ampicillin, gentamycin, and metronidazole two days post-surgery, followed by oral antibiotics. Acetaminophen and ibuprofen were prescribed for pain relief. The patient reported regular voiding four hours post-surgery and immediate pain relief was observed. Additionally, she was advised to apply ice packs and educated on perineum care and hygiene. Six weeks of pelvic rest was recommended. The patient experienced a smooth recovery.

She was followed up by a midwife in the sixth and 10 weeks after the surgery at a local clinic. No complaints were reported during these visits. The drain was removed after one week, and the patient was advised to return in case of any signs of pain, fever, swelling, bleeding, or foul-smelling discharge from the wound or vagina.

## Discussion

Vulvar hematomas in non-obstetric women are infrequent and have not been widely documented in the literature. The reported incidence of these hematomas is approximately 3.7%, accounting for up to 0.8% of all admissions related to gynecologic cases [6]. In cases of vulvar hematomas, the bleeding is confined to the area above the anterior urogenital diaphragm. On the other hand, in vulvovaginal hematomas, the bleeding extends to the surrounding paravaginal tissues [7]. The vulva consists of loose connective tissue and smooth muscle, which receives blood supply from branches of the pudendal artery, which arises from the internal iliac artery [8]. Injuries to the labial branches of the internal pudendal artery, located within the superficial fascia of the anterior and posterior pelvic triangle, can result in significant vulvar hematomas [9].

These hematomas are caused by blunt trauma that forces the underlying pelvic fascia against the pelvic bones, leading to laceration and hematoma formation. In such cases, the swelling tends to follow cleavage planes and can grow in size due to the low resistance offered by the subcutaneous tissue [10]. If bleeding occurs beneath the pelvic fascia and the levator ani, it can separate the latter from the perineum. Alternatively, if the hematoma forms on the pelvic fascia, it can extend below Poupart's ligament and continue retroperitoneally to the renal fossae [11].

A case report has highlighted an unusual occurrence of a vulvar hematoma measuring 15 cm, possibly resulting from the rupture of a pseudoaneurysm in the left pudendal artery in the absence of any traumatic injury [12]. In another report, following a straddle injury, a 16-year-old patient presented with a vulvar hematoma attributed to coagulopathy resulting from a rickettsial infection [13]. Very few studies have mentioned the postoperative complication of vulvar hematoma after radical vulvectomy for vulvar cancer and clitoral reconstruction for genital mutilation [14,15].

Perineal ultrasonography, CT, and MRI play a crucial role in diagnosing the magnitude of hematomas, as well as identifying vascular and bony injuries within the pelvic region [12]. According to a study, surgical evacuation of hematomas can be conducted when there is a large hematoma (average size: 9.5 cm), severe pain, distorted vulvar anatomy, and difficulty locating the urethral opening for inserting an indwelling urinary catheter. Furthermore, it has been suggested that surgical management has the potential to accelerate the recovery process [16].

The conventional treatment options for traumatic vulvar hematomas include conservative management and surgical intervention. When there is no significant expansion of an acute hematoma, conservative management is often effective [1]. In a case report of a patient with non-obstetric vulvar hematoma, the patient underwent transarterial embolization of the left internal pudendal artery [17]. Another study has mentioned performing a vaginal incision to drain a large hematoma to prevent scarring in the vulva region [3]. In innovative treatment approaches, a vaginal incision for draining vulvar hematomas and performing pudendal artery embolization have shown promising esthetic results, particularly in well-equipped healthcare settings. The conservative and surgical treatment approach should be tailored to the specific clinical presentation, based on whether the patient presents in an emergent or non-emergent situation.

The scarcity of literature on the treatment modalities of non-obstetric vulvar hematomas can be attributed to the limited number of reported cases and the absence of standardized practice guidelines [3]. Hence, there is a need for further research in this field.

## Conclusions

Delayed diagnosis or treatment of a significant traumatic vulvar hematoma can lead to risks of severe morbidity and mortality. In low-resource settings, prompt intervention significantly minimizes the chances of skin necrosis, infection, abscess formation, and prolonged hospitalization. The treatment approach is determined by the patient's clinical presentation. Further research is necessary to establish comprehensive treatment guidelines that are effective and evidence-based.
